# Activation of MT2 receptor ameliorates dendritic abnormalities in Alzheimer’s disease via C/EBPα/miR‐125b pathway

**DOI:** 10.1111/acel.12902

**Published:** 2019-02-01

**Authors:** Hui Tang, Mei Ma, Ying Wu, Man‐Fei Deng, Fan Hu, Hasan.a.m.m. Almansoub, He‐Zhou Huang, Ding‐Qi Wang, Lan‐Ting Zhou, Na Wei, Hengye Man, Youming Lu, Dan Liu, Ling‐Qiang Zhu

**Affiliations:** ^1^ Department of Pathophysiology, Key Lab of Neurological Disorder of Education Ministry, School of Basic Medicine, Tongji Medical College Huazhong University of Science and Technology Wuhan China; ^2^ The Institute of Brain Research, Collaborative Innovation Center for Brain Science Huazhong University of Science and Technology Wuhan China; ^3^ Department of Pathology The First Affiliated Hospital of Zhengzhou University Zhengzhou China; ^4^ Department of Pathology, School of Basic Medicine Zhengzhou University Zhengzhou China; ^5^ Department of Biology Boston University Boston Massachusetts; ^6^ Department of Genetics, School of Basic Medicine, Tongji Medical College Huazhong University of Science and Technology Wuhan China

**Keywords:** Alzheimer’s disease, dendritic complexity, dendritic spines, melatonin, miRNA, MT2

## Abstract

Impairments of dendritic trees and spines have been found in many neurodegenerative diseases, including Alzheimer's disease (AD), in which the deficits of melatonin signal pathway were reported. Melatonin receptor 2 (MT2) is widely expressed in the hippocampus and mediates the biological functions of melatonin. It is known that melatonin application is protective to dendritic abnormalities in AD. However, whether MT2 is involved in the neuroprotection and the underlying mechanisms are not clear. Here, we first found that MT2 is dramatically reduced in the dendritic compartment upon the insult of oligomer Aβ. MT2 activation prevented the Aβ‐induced disruption of dendritic complexity and spine. Importantly, activation of MT2 decreased cAMP, which in turn inactivated transcriptional factor CCAAT/enhancer‐binding protein α(C/EBPα) to suppress miR‐125b expression and elevate the expression of its target, GluN2A. In addition, miR‐125b mimics fully blocked the protective effects of MT2 activation on dendritic trees and spines. Finally, injection of a lentivirus containing a miR‐125b sponge into the hippocampus of APP/PS1 mice effectively rescued the dendritic abnormalities and learning/memory impairments. Our data demonstrated that the cAMP‐C/EBPα/miR‐125b/GluN2A signaling pathway is important to the neuroprotective effects of MT2 activation in Aβ‐induced dendritic injuries and learning/memory disorders, providing a novel therapeutic target for the treatment of AD synaptopathy.

## 1 INTRODUCTION

Alzheimer's disease (AD) is one of the most prevalent neurodegenerative diseases in aged people, which is characterized by the progressive memory decline clinically. Synaptic disorder had been implicated in the memory impairment of AD during the early stage. Generally, the synapse is composed of presynaptic parts and postsynaptic parts, the latter one relies on the proper development of dendritic complexity and spines. Previous studies had reported that the dendritic abnormalities, including the spine loss, shaft atrophy, dendritic bending, and reduction of the dendritic complexity, were found in the neocortex and hippocampus of both the AD mouse model and human brains (Grutzendler, Helmin, Tsai, & Gan, 2007; Guo et al., 2017) at early stage. In AD, a negative linear correlation between reduction of the dendritic complexity and Aβ deposition had been found, which hints that Aβ deposition could be the primary event in generating various dendritic abnormalities.

Melatonin is a hormone that is dominantly secreted from the pineal gland and possesses multiple physiological functions, such as circadian rhythm regulation, free radical clearance, immune modulation, and biomolecule oxidation suppression (Reiter et al., 2016). Melatonin also protects neurons from different stimuli, especially in neurodegenerative diseases (Miller, Morel, Saso, & Saluk, 2015; Ng, Leong, Liang, & Paxinos, 2017). For example, in AD, the level of melatonin and its membranous receptor melatonin receptor 2 (MT2) is reduced in the brain (Brunner et al., 2006; Savaskan et al., 2005), and melatonin supplementation restores synaptic disorder and memory deficits in Aβ‐treated rats (Liu et al., 2013) and a transgenic AD mouse model (Peng et al., 2013). In aged rats, long‐term melatonin treatment attenuates the decrease in dendritic protein MAP‐2 immunostaining and improves dendritic stability, thus diminishing synaptic elimination (Prieto‐Gomez et al., 2008). Acute melatonin treatment increases apical dendritic length and dendritic complexity in the CA1 region (Ikeno & Nelson, 2015). In our previous study, we also found that melatonin treatment rescues the dendritic spine impairments induced by scopolamine (Wang et al., 2013). Thus, melatonin treatment might be a useful approach to restore dendritic spine injury. Most of the biological effects of melatonin are known to be mediated by its membranous receptors, MT1 and MT2 (Liu et al., 2016). MT2 is mainly distributed in the hippocampus (Lacoste et al., 2015), a critical brain region in memory processes and synaptic plasticity. However, how MT2 is involved in the protection of dendritic complexity and spines by melatonin remains unclear.

In this study, we examined the possible protective effects of MT2 activation on the dendritic impairments induced by oligomeric Aβ42‐amyloid protein (Aβ42). We found that oligomeric Aβ42 insult reduces the expression of MT2 and impairs dendritic morphology. Activation of MT2 by selective and nonselective agonists prevents or ameliorates the dendritic injuries caused by Aβ42. Finally, we found that the C/EBPα/miR‐125b signal mediates the protective effect of MT2 activation on dendritic injuries. Our data here provide the basic evidence for the role of MT2 activation in dendritic protection in AD.

## 2 RESULTS

### 2.1 MT2 expression is decreased in AD models

**Figure 1:**
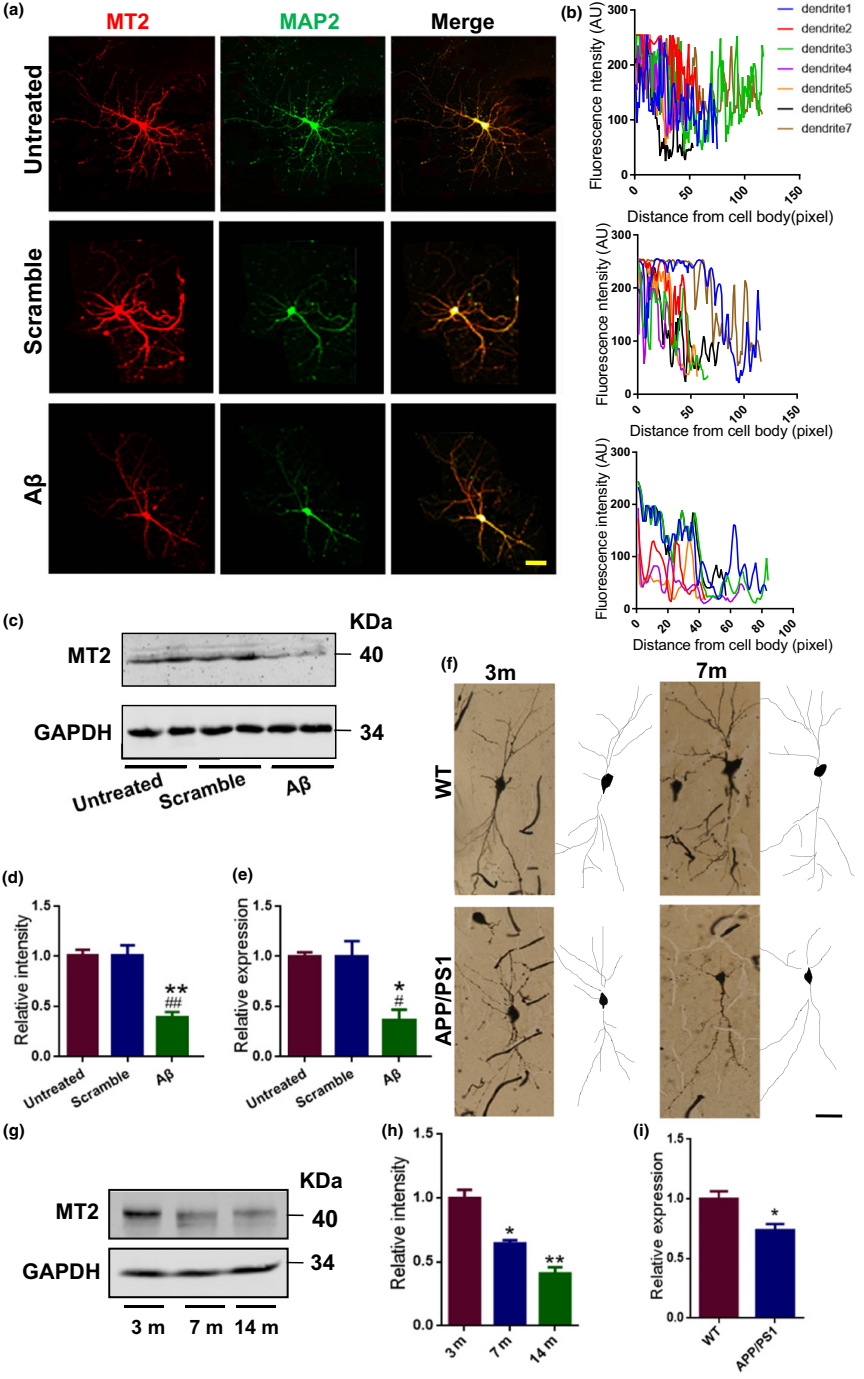
MT2 expression is reduced in AD model both in vivo and in vitro. (a–e) Mouse primary hippocampal neurons treated with 1 μM oligomeric Aβ and its scrambled peptide at DIV 7 for 2 days. The neurons (DIV 9) treated or left untreated were fixed with 4% PFA and costained with anti‐MT2 (red) and anti‐MAP2 (green) antibodies. Representative confocal images are shown (a). Scale bar = 40 μm. MT2 immunofluorescence intensity in different neuronal dendrites when neurons were treated with (b, lowest panel), without Aβ42 (b, middle panel), or left untreated (b, top panel). AU means arbitrary unit. Cell lysates from neurons treated with Aβ, the scrambled, or left untreated were evaluated by Western blot with an anti‐MT2 antibody (c), and the MT2 expression level was quantified (d). Total RNA was extracted, followed by reverse transcription as described previously. Relative MT2 mRNA expression from the above cDNA was quantified by real‐time PCR (e). *p < 0.05, **p < 0.01 (vs. Untreated); ^#^ *p* < 0.05, ^##^ *p* < 0.01 (vs. Scramble), one‐way ANOVA, Tukey's multiple comparisons test). *N* = 4–5. All the experiments were repeated by at least three times. Data are presented as the mean ± *SEM*. (f) Golgi staining was performed on APP/PS1 mice and their WT littermates to evaluate the dendritic morphology at 3 and 7 months of age. Representative Golgi staining image (left) and reconstructions (right) are shown. Scale bar = 40 μm. *N* = 4–5 mice per group. (g–h) Hippocampal homogenates were prepared from 3‐, 7‐, and 14‐month‐old APP/PS1‐AD mice (Tg); then, the MT2 protein level was examined by Western blot. Representative images (g) and the quantitative analysis (h) are presented. *p < 0.05, **p < 0.01 (vs. 3 m); one‐way ANOVA, Tukey's multiple comparisons test; *N* = 4–5 mice per group. All the experiments were repeated by at least three times. (i) Relative expression level of MT2 mRNA in 7‐month‐old APP/PS1 and WT mouse hippocampus homogenates by qPCR assay. *p < 0.05 (vs. WT); Student's *t* test; *N* = 4–5 mice per group

In AD, impaired dendritic trees and spines are recognized as the hallmarks of synaptic dysfunction (van Wijk et al., 2014), and the Aβ42 oligomer is the primary toxin that induces synaptic disorder. We then examined whether Aβ42 affects the expression of MT2 in dendrites. We administered 1 μM oligomeric form Aβ42 (Supporting Information Figure S1) to DIV 7 hippocampal neurons and collected the lysate for Western blotting and RT–PCR at 48 hr later. We found that the immunoreactivity of MT2 in the dendritic compartment was suppressed by Aβ42 treatment when compared to the scrambled peptide‐treated neurons or the untreated neurons (Figure 1a–b), suggesting a reduction in MT2 expression in dendrites upon Aβ42 insults in vitro. Meanwhile, the protein and mRNA levels of MT2 in oligomeric Aβ‐treated neurons decreased to 38.63% and 36.24% of the levels in scrambled peptide‐treated neurons and 38.45% and 36.34% of the levels in untreated neurons, respectively (Figure 1c–e). Concomitantly, no significant neuron loss was detected upon Aβ treatment (Supporting Information Figure S2). Furthermore, in APP/PS1 mice, a widely used AD mouse model, MT2 protein and mRNA expression levels were dramatically reduced in the hippocampus beginning at 7 months of age (Figure 1f–i), at which point dendritic abnormalities were also found (Figure 1f). These data suggested that MT2 expression is decreased in AD models.

### 2.2 Activation of MT2 rescues the dendritic impairments induced by Aβ

**Figure 2:**
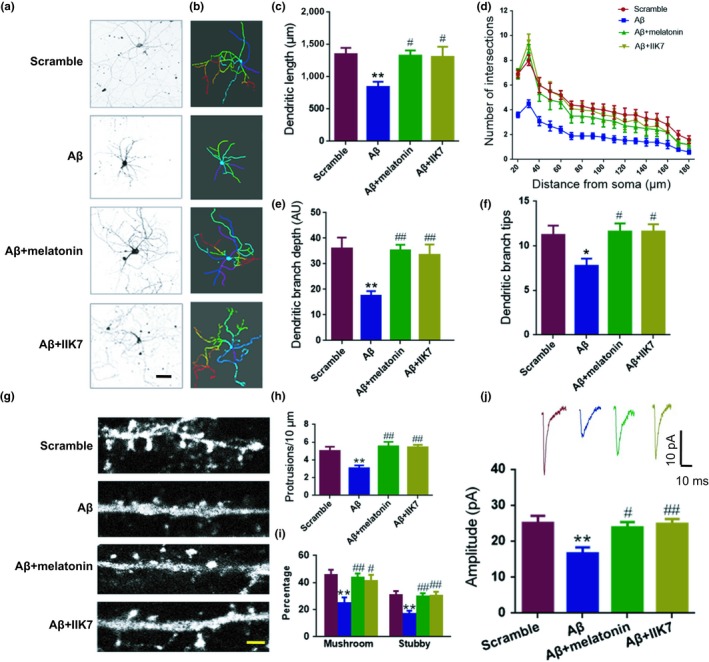
Activation of the MT2 receptor rescues the Aβ42‐induced dendritic impairment. (a–f) Mouse primary hippocampal neurons at DIV 7 were treated with Aβ, Aβ + melatonin, Aβ + IIK7, or the scrambled peptide for 48 hr. Representative images after treatment (a) and 3D reconstructions of neurons in panel (a) are shown in (b), color‐coded is according to their branch depth. Warmer hues indicate higher branch depth or tip orders. AU means arbitrary unit. Scale bar = 40 μm. (c–f) Quantitative analyses of dendritic length (μm) (c), Sholl analysis (d), dendritic branch depth (e), and dendritic branch tips (f) were conducted by Imaris software. *p < 0.05, **p < 0.01 (vs. Scramble; one‐way ANOVA, Tukey's multiple comparisons test). ^#^ *p* < 0.05, ^##^ *p* < 0.01 (vs. Aβ; one‐way ANOVA, Tukey's multiple comparisons test). *N* = 13–15. Data are presented as the mean ± *SEM*. (g–j) Mouse primary hippocampal neurons treated with Aβ42, Aβ42 + melatonin, Aβ42 + IIK7, or the control scrambled Aβ42 peptide at DIV 19 were cultured to DIV 21. (g) Representative confocal micrographs are shown. The spines were visualized using GFP‐expressing lentivirus. Scale bar=3 μm. Then, spine density (protrusions/10 μm) (h) and percentage of mushroom and stubby‐like spines (i) were analyzed. *N* = 13–15. (j) Whole‐cell patch clamp recording was performed as described in the “Materials and Methods” section. Representative mEPSC curve (upper panel) and quantitative analysis of amplitude (pA) (lower panel) are presented. **p < 0.01 (vs. Scramble; one‐way ANOVA, Tukey's multiple comparisons test). ^#^ *p* < 0.05, ^##^ *p* < 0.01 (vs. Aβ; one‐way ANOVA, Tukey's multiple comparisons test). *N* = 8–10 slice from 4 to 5 mice for each group. Data are presented as the mean ± *SEM*

We then asked whether activation of the remaining MT2 receptor could rescue the Aβ‐induced dendritic impairments. At DIV 7, we treated cultured neurons with 1 μM oligomeric Aβ42 plus melatonin or IIK7 (a selective agonist with 90‐fold higher affinity for MT2) (Alarma‐Estrany, Crooke, Mediero, Pelaez, & Pintor, 2008; Liu et al., 2015) or the respective controls. At DIV 9, anti‐MAP2 antibody was used to examine dendritic morphology. Compared to the scrambled peptide, 1 μM Aβ42 alone caused obvious impairments in the dendritic complexity at all points farther than 40 µm from the cell body when compared with scrambled peptide (Figure 2a–f), which is consistent with previous studies (Wang et al., 2018). Co‐application of melatonin or IIK7 effectively rescued the dendritic complexity damage caused by Aβ42 (Figure 2a–f). We also treated the cultured neurons with 1 μM Aβ42 plus melatonin or IIK7 and the respective controls at DIV 19 and assayed the changes in dendritic spines at DIV 21. We found that Aβ42 significantly reduced the density of spines, percent of mushroom, and stubby‐like spines, while melatonin and IIK7 restored those abnormalities (Figure 2g–i). Concomitantly, melatonin and IIK7 effectively rescued the Aβ‐induced reduction in mini‐excitatory postsynaptic current (mEPSC) amplitude (Figure 2j). Importantly, all those treatments did not alter the expression of MT1 receptor (Supporting Information Figure S3), which is consistent with a previous study (Cecon et al., 2015). These data strongly suggested that activation of MT2 protects against the dendritic impairments caused by oligomeric Aβ.

### 2.3 cAMP‐C/EBPα/miR‐125b signaling mediates the neuroprotective effects of MT2 activation on Aβ‐induced dendritic abnormalities

**Figure 3:**
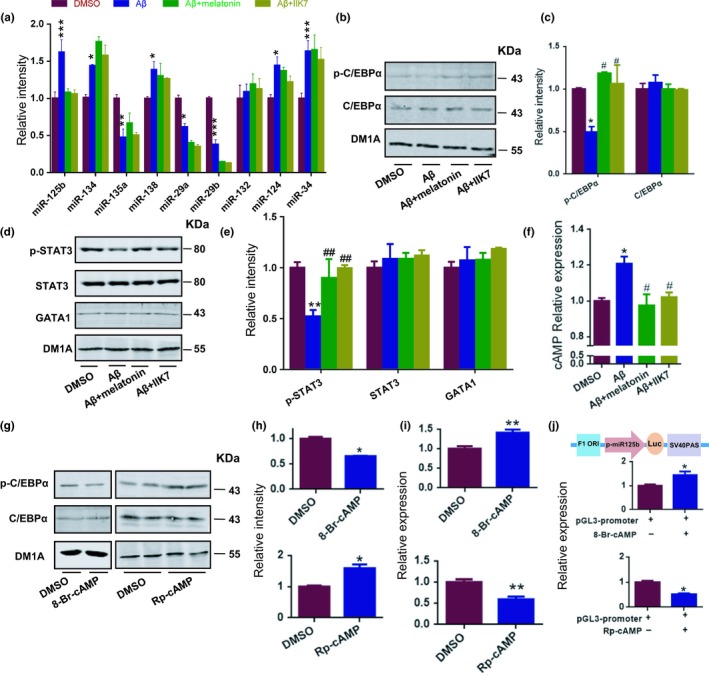
The neuroprotective effect of MT2 activation is mediated by cAMP‐C/EBPα‐miR‐125b signaling. (a–f) Mouse primary hippocampal neurons at DIV 7 were treated with Aβ, Aβ + melatonin, Aβ + IIK7, or the scrambled peptide for 2 days. (a) Total RNA was extracted from mouse primary hippocampal neurons at DIV 9 and reverse transcribed by using a miRcute miRNA first‐strand cDNA Synthesis Kit (Tiangen, Inc.). The levels of miR‐125b, miR‐134, miR‐135a, miR‐138, miR‐29a/b, miR‐132, miR‐124, and miR‐34 were quantified by qPCR. *N* = 4–5. (b–g) The nuclear extracts from the neurons above were evaluated by Western blot with the anti‐pSer21‐C/EBPα antibody (p‐C/EBPα), anti‐C/EBPα antibody (b), anti‐pY705‐STAT3 antibody (pY705‐STAT3), anti‐STAT3 antibody, and anti‐GATA1 antibody (d). Quantitative analyses of panel (b) and panel (d) are shown in panel (c) and panel (e), respectively. *N* = 4–5. (f) cAMP production level in the neurons above was quantified with an ELISA kit. *p < 0.05, **p < 0.01, ***p < 0.001 (vs. DMSO; one‐way ANOVA, Tukey's multiple comparisons test). ^#^ *p* < 0.05, ^##^ *p* < 0.01 (vs. Aβ; one‐way ANOVA, Tukey's multiple comparisons test). *N* = 4–5. All the experiments were repeated by at least three times. Data are presented as the mean ± *SEM*. (g–i) Mouse primary hippocampal neurons at DIV 7 were treated with 8‐Br‐cAMP as well as Rp‐cAMP. One hour later, nuclear lysates were prepared and quantified via Western blot assay with the anti‐pSer21‐C/EBPα antibody (p‐C/EBPα) and anti‐C/EBPα antibody (g). Quantitative analyses are shown in panel (h). (i) Relative expression of miR‐125b after 8‐Br‐cAMP and Rp‐cAMP treatment. *p < 0.05, **p < 0.01 (vs. DMSO; Student's *t* test). *N* = 4–5. All the experiments were repeated by at least three times. Data are presented as the mean ± SEM. (j) The promoter region of miR‐125b was cloned into the pGL3 vector (pGL3‐miR‐125b). The HEK293 cells were transfected with pGL3‐miR‐125b. Forty‐seven hours later, the cells were treated with 8‐Br‐cAMP and Rp‐cAMP for 1 hr. Then, cell lysates were collected and analyzed with firefly luciferase assay. Upper panel is the diagram of pGL3‐miR‐125b plasmid. p‐miR‐125b, promoter region of miR‐125b; SV40 PAS, SV40 poly A signal. *p < 0.05 (vs. control; Student's *t* test). *N* = 4–6. All the experiments were repeated by at least three times. Data are presented as the mean ± *SEM*

Next, we examined the exact underlying mechanisms of MT2’s protective effects against Aβ42‐induced dendritic injuries. Recently, the roles of microRNAs (miRNAs) in synaptic disorder observed in AD have been well elucidated (Li et al., 2018; Liu et al., 2017; Wang et al., 2018). Therefore, we then measured the expression of brain‐enriched miRNAs that had been reported previously upon the different treatments above. We found that Aβ42 treatment disrupted the expression of multiple miRNAs, including the upregulation of miR‐125b, miR‐134, miR‐138, miR‐124, and miR‐34, downregulation of miR‐135a and miR‐29a/b, and the unalteration of miR‐132 (Figure 3a). However, co‐administration of melatonin or IIK7 only corrected the maladjustments of miR‐125b but not the other miRNAs (Figure 3a), suggesting that miR‐125b may be involved in the MT2‐mediated protection against dendritic abnormalities. We then explored how MT2 could regulate the expression of miR‐125b. Previous studies have revealed that the level of miR‐125b is controlled by the transcriptional factors STAT3 (Liu et al., 2011), CCAAT/enhancer‐binding proteinα (C/EBPα) (Liu et al., 2011; Vargas et al., 2015), CDX2 (Lin et al., 2011), and GATA1 (Lin et al., 2011) (Supporting Information Table S3). Considering that CDX2 is not distributed in the brain according to the brain atlas (http://www.proteinatlas.org/ENSG00000165556-CDX2/tissue), we then examined the activity of the other transcriptional factors upon Aβ42 stimulation with or without MT2 activation by melatonin or IIK7 in DIV 7 hippocampal neurons. Western blot analysis indicated that the Aβ insult did not change the total protein level of C/EBPα. However, by detecting the inhibitory phosphorylation of C/EBPα at Ser 21, we found that Ser 21‐phosphorylated C/EBPα (p‐C/EBPα) expression was significantly decreased by Aβ42 treatment, indicating that the Aβ insult significantly elevated C/EBPα activity, which was suppressed by co‐application with melatonin or IIK7 (demonstrated by the inhibitory phosphorylation of Ser 21) (Figure 3b–c). In addition, Aβ treatment had no impact on the expression level of GATA1 (Figure 3d–e). However, STAT3 activity (demonstrated by the phosphorylation of STAT3 at Tyr 705 site), not the total protein level of STAT3, was significantly downregulated upon Aβ42 insult (Figure 3d–e), and co‐application with melatonin or IIK7 increased STAT3 activity (Figure 3d–e), which is also verified by the expression of c‐fos (Supporting Information Figure S4), a well‐known downstream molecule of STAT3 (Carpenter & Lo, 2014). Considering the positive transcriptional regulation of STAT3 and C/EBPα on miR‐125b, we concluded that MT2 activation restores the expression of miR‐125b by inhibiting C/EBPα but not STAT3. In addition, silencing of C/EBPα with specific shRNA plasmid restored miR‐125b level that elevated by Aβ42 insult (Supporting Information Figure S5a–c).

MT2 activation is known to induce cyclic adenosine monophosphate (cAMP) inhibition (Petit, Lacroix, de Coppet, Strosberg, & Jockers, 1999), and cAMP has been reported to inhibit dendrite growth (Yamada, Matsuki, & Ikegaya, 2005). Therefore, we examined the concentration of cAMP upon different treatments. In line with other reports (Igbavboa et al., 2006; Miller, Isenberg, Shih, Wang, & Roberts, 2010), we found that Aβ42 treatment elevated the concentration of cAMP (Figure 3f). In contrast, both melatonin and IIK7 suppressed cAMP expression (Figure 3f). We then examined the potential effects of cAMP signal regulation on the activity of C/EBPα by treating primary neurons with 2 mM 8‐Br‐cAMP (a cAMP analog), 100 μM Rp‐cAMP (a cAMP inhibitor), or vehicle. We found that C/EBPα activity was activated upon 8‐Br‐cAMP treatment but inhibited by Rp‐cAMP (Figure 3g–h). Furthermore, 8‐Br‐cAMP treatment increased miR‐125b expression, whereas Rp‐cAMP suppressed it (Figure 3i). In HEK293 cells transfected with a luciferase reporter under the miR‐125b promoter, 8‐Br‐cAMP stimulated but Rp‐cAMP reduced the luciferase intensity (Figure 3j). These data suggested that the cAMP‐C/EBPα signal plays an important role in the neuroprotective effects of MT2 activation against Aβ42‐induced dendritic abnormalities.

**Figure 4:**
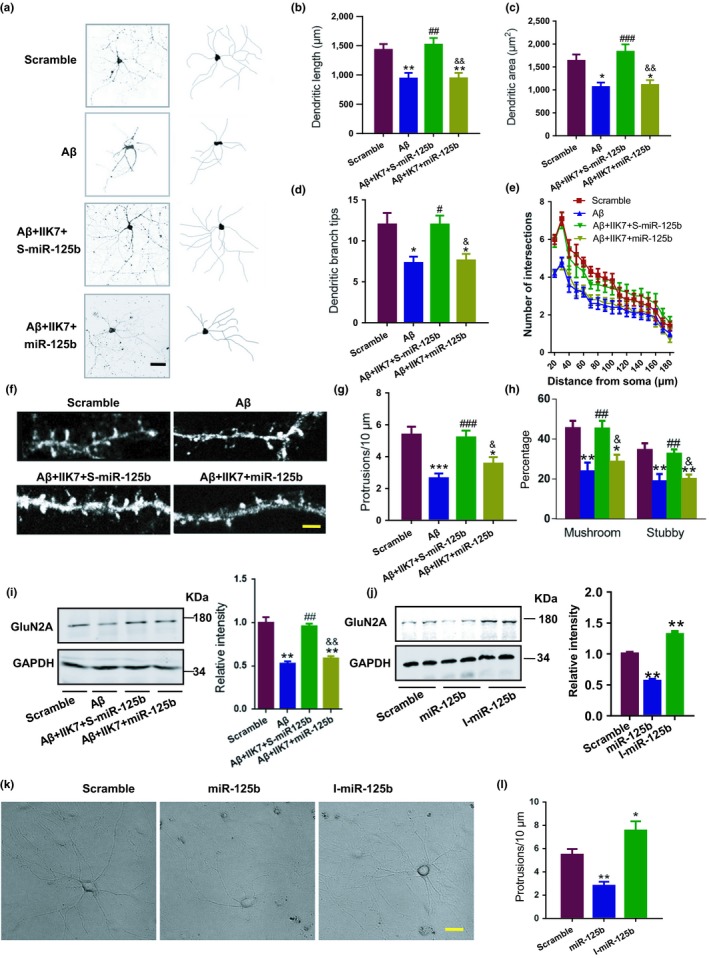
MiR‐125b suppression is involved in the protective effects of MT2 on dendritic impairments. (a–e) Mouse primary hippocampal neurons at DIV 7 were treated with scrambled peptide (Scramble), Aβ, Aβ plus IIK7 plus the scrambled control for miR‐125b (Aβ + IIK7 + S‐miR‐125b) or Aβ plus IIK7 plus miR‐125b mimics (Aβ + IIK7 + miR‐125b) for 2 days. (a) Representative confocal images (a, left) and their reconstructions (a, right) are shown. Scale bar = 40 μm. Quantitative analysis of total dendritic length (μm) (b), dendritic area (μm^2^) (c), dendritic branch tips (d), and number of intersections (e) are presented. *p < 0.05, **p < 0.01 (vs. Scramble; one‐way ANOVA, Tukey's multiple comparisons test). ^#^ *p* < 0.05, ^##^ *p* < 0.01, ^###^ *p* < 0.001 (vs. Aβ; one‐way ANOVA). ^&^ *p* < 0.05, ^&&^ *p* < 0.01 (vs. Aβ + IIK7 + S‐miR‐125b; one‐way ANOVA, Tukey's multiple comparisons test). *N* = 12–15. Data are presented as the mean ± *SEM*. (f–i) Mouse primary hippocampal neurons at DIV 19 were treated with the scrambled Aβ42 peptide (Scramble), Aβ, Aβ + IIK7 + S‐miR‐125b or Aβ + IIK7 + miR‐125b and cultured to DIV 21. Representative confocal images (f), quantitative analysis of spine density (protrusions/10 μm) (g), and the percentage of mushroom and stubby‐like spines (h) are shown. Scale bar = 2 μm. *N* = 12–14. (i) Cell lysates were collected from the neurons treated as described in (f), and Western blot was used to evaluate the protein level of GluN2A. *N* = 4–5. All the experiments were repeated by at least three times. Representative images (left panel) and the quantitative analysis (right panel) are shown. *p < 0.05, **p < 0.01 (vs. scramble; one‐way ANOVA, Tukey's multiple comparisons test). ^#^ *p* < 0.05, ^##^ *p* < 0.01, ^###^ *p* < 0.001 (vs. Aβ; one‐way ANOVA, Tukey's multiple comparisons test). ^&^ *p* < 0.05, ^&&^ *p* < 0.01 (vs. Aβ + IIK7 + S‐miR‐125b; one‐way ANOVA, Tukey's multiple comparisons test). Data are presented as the mean ± *SEM*. (j) Cell lysates from DIV 21 primary hippocampal neurons treated with miR‐125b, the inhibitor of miR‐125b (I‐miR‐125b), and the scrambled control (Scramble) were collected. Western blot was used to evaluate the protein level of GluN2A. **p < 0.01 (vs. Scramble; one‐way ANOVA, Tukey's multiple comparisons test). *N* = 4–5. All the experiments were repeated by at least three times. (k) Representative images of primary hippocampal neurons treated as described in (j) are shown. Scale bar = 20 μm. Quantitative analyses of spine density (protrusions/10 μm) were performed (l). *p < 0.05, **p < 0.01 (vs. Scramble; one‐way ANOVA, Tukey's multiple comparisons test). *N* = 11–15. Data are presented as the mean ± *SEM*

Then, we asked whether miR‐125b participates in the protective effects of MT2 activation in dendrites. We found that application of miR‐125b mimics not only enhanced the expression of miR‐125b from 1 nM (Supporting Information Figure S6), but also apparently abolished the IIK7‐induced amelioration of the dendritic complexity impairment (Figure 4a–e) and spine disorder (Figure 4f–h). Furthermore, IIK7 application restored the protein expression of GluN2A (Figure 4i), a well‐known target of miR‐125b (Figure 4j, Supporting Information Figure S7), while miR‐125b mimics hindered this restoration (Figure 4i). In addition, introduction of GluN2A into miR‐125b mimic treated wells could rescue miR‐125b induced dendritic complexity impairment (Supporting Information Figure S8), which indicate that miR‐125b mimic disrupting dendrite integrity in a GluN2A‐dependent fashion.

These data verified that the protective effects of MT2 activation require the suppression of miR‐125b expression. In addition, manipulating the miR‐125b expression (Supporting Information Figure S9a) alone induced dendritic complexity decrement (Figure 4k) and spine abnormalities (Figure 4l) directly.

### 2.4 Inhibition of miR‐125b rescues the dendritic abnormalities and learning/memory impairments in AD mice

**Figure 5:**
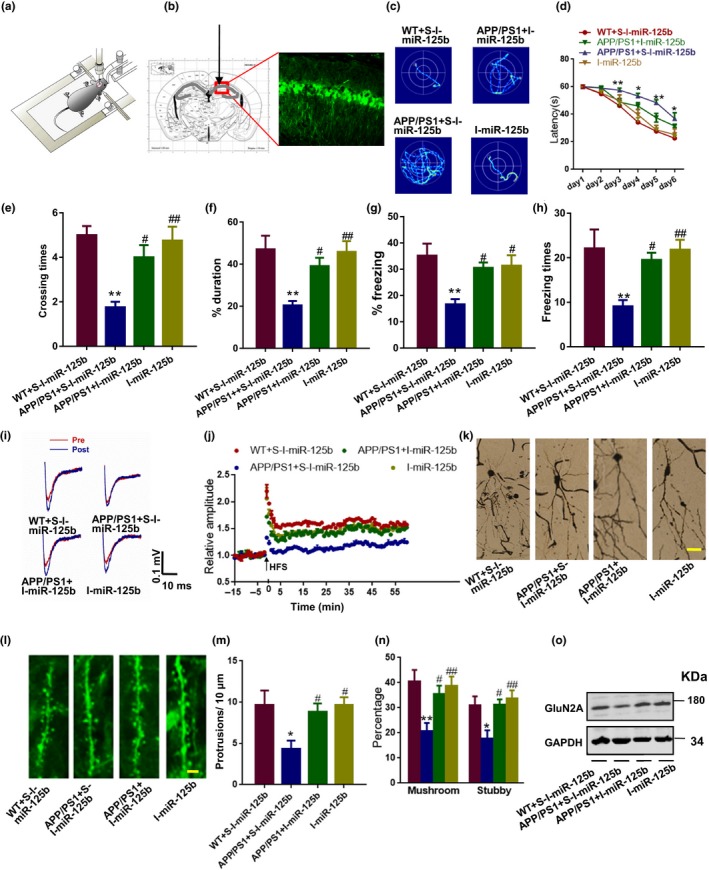
Inhibition of miR‐125b rescues the dendritic abnormalities and learning/memory impairments in AD mice. (a–h) The CA1 of 5‐month‐old APP/PS1 mice and WT littermates was injected with I‐miR‐125b or its scrambled control as indicated. Then, 1 month later, the mice were subjected to the Morris water maze and context fear conditioning test to evaluate learning and memory. The diagram for the stereotactic injection experiment is shown in (a), and a representative image of the injection site and virus expression is shown in (b). (c–f) Representative traces (c) and latencies (d) to the platform were recorded during final training day (day 7). *F*_3, 15_ = 8.342. *p < 0.05, **p < 0.01 (vs. WT + S‐I‐miR‐125b mice; two‐way ANOVA, *N* = 6–8 for each group). Data are presented as the mean ± *SEM*. On the ninth day of the Morris water maze test, the platform was removed, and the probe test was performed. The crossing times to the platform region (e) and the percentage of duration spent in the target quadrant (f) were analyzed. *p < 0.05, **p < 0.01 (vs. WT + S‐I‐miR‐125b mice); ^#^ *p* < 0.05, ^##^ *p* < 0.01 (vs. APP/PS1 + S‐I‐miR‐125b mice); one‐way ANOVA, Tukey's multiple comparisons test, *N* = 6–8 for each group. Data are presented as the mean ± *SEM*. (g–h) Mice were also subjected to the context fear conditioning test. The freezing response during a retrieval test performed 24 hr after fear training (recent memory) was analyzed. Percentage of time spent freezing (g) and number of freezing bouts (h) were quantified. **p < 0.01 (vs. WT + S‐I‐miR‐125b); ^#^ *p* < 0.05, ^##^ *p* < 0.01 (vs. APP/PS1 + S‐I‐miR‐125b); one‐way ANOVA, Tukey's multiple comparisons test. *N* = 6–8 for each group. Data are presented as the mean ± *SEM*. (i–j) Brain slices at 300 μm thick were collected and used for the MED64 electrophysiological recording. Representative field excitatory postsynaptic potential (fEPSP) traces (red for pre‐high‐frequency stimulation (HFS) and blue for post‐HFS) (left panel) and the quantitative analysis for the 75‐min recording (right panel) (i). Time courses of mean fEPSP slope changes in response to HFS of all activated channels in 6–7 slices from three mice each for WT + S‐I‐miR‐125b, APP/PS1 + S‐I‐miR‐125b, APP/PS1 + I‐miR‐125b, and I‐miR‐125b groups (j). (k–n) Brain slices were collected for dendritic morphology and for the evaluation of dendritic spines. Representative images of dendrites (k) and dendritic spines (l) are shown. Scale bar = 40 μm for (k); Scale bar = 3 μm for (l). Quantitative analyses of the spine density (m) and the percentage of mushroom and stubby‐like spines (n) were performed. *p < 0.05, **p < 0.01 (vs. WT + S‐I‐miR‐125b); ^#^ *p* < 0.05, ^##^ *p* < 0.01 (vs. APP/PS1 + S‐I‐miR‐125b); one‐way ANOVA, Tukey's multiple comparisons test. *N* = 8–10 slice from 3 to 4 mice for each group. Data are presented as the mean ± *SEM*. (o) Hippocampal tissue lysates from the mice above were collected, and Western blot was used to detect the protein level of GluN2A. *N* = 4–5. All the experiments were repeated by at least three times

Finally, we examined whether inhibiting upregulated miR‐125b in vivo could rescue the dendritic abnormalities and learning/memory impairments in AD mice, in which the MT2 signal is downregulated. We injected a verified recombinant lentivirus‐packaged sponge for miR‐125b (I‐miR‐125b) into the hippocampal CA1 area of APP/PS1 mice at 5 months of age (Figure 5a–b). The sponge is effective because seed‐site mutant control sponge did not alter the level of miR‐125b at all (Supporting Information Figure S9b). We found that the spatial learning impairments exhibited by APP/PS1 mice in the Morris water maze task were rescued by I‐miR‐125b treatment beginning on the third day (Figure 5c,d). In the probe trial, the crossing times and duration spent in the target quadrant were also recovered by I‐miR‐125b treatment (Figure 5e,f). In a context fear conditioning test, inhibition of miR‐125b improved both the percentage of freezing duration and freezing times (Figure 5g,h). Electrophysiological recording from CA3‐CA1 projection indicated that the deficits in long‐term potentiation (LTP) were also attenuated by I‐miR‐125b treatment (Figure 5i,j). Importantly, the abnormalities in the dendritic complexity (Figure 5k) and spines (Figure 5l–n) in the CA1 pyramidal neurons, as well as the loss of GluN2A (Figure 5o), were rectified after I‐miR‐125b treatment. These data strongly suggested that inhibition of miR‐125b, the downstream effector of MT2 activation, is able to rescue the dendritic abnormalities and memory impairments observed in AD.

## 3 DISCUSSION

In the nervous system, the characteristic dendritic branching and the extension of these dendritic branches into specific spatial domains are the primary contributors to the connectivity between neurons. In addition to complicated arbors, dendrites also develop small protrusions termed dendritic spines to act as the postsynaptic site, receiving signals from axonal terminals. Since Cajal first depicted the tree‐like morphology of dendrites and filopodia‐like dendritic spines, many molecules have been found to be essential for the development of dendritic tree and spines (O'Donnell, Chance, & Bashaw, 2009), including many membranous receptors, one of which is a receptor for melatonin, a hormone that is able to promote dendrite maturation and complexity in adult mouse hippocampal neurogenesis (Ramirez‐Rodriguez, Ortiz‐Lopez, Dominguez‐Alonso, Benitez‐King, & Kempermann, 2011). MT2 is enriched in hippocampal neurons both in vivo and in vitro, suggesting a critical role of MT2 in the normal function of hippocampus. We found here that the MT2 level is dramatically decreased upon the stimulation of Aβ and in the hippocampus of APP/PS1 mice, which is consistent with a previous report (Savaskan et al., 2005). Moreover, we identified that the loss of MT2 in cultured neuron is most significant in the somatodendritic compartment. This raised our interest to explore the potential protective role of MT2 in dendritic abnormalities in AD.

The integrity of dendritic trees and the density of spines are important for synaptic plasticity, and disruption of dendritic morphology had been found in many neurodegenerative diseases associated with memory deficits, such as AD. AD is characterized by two pathological hallmarks, extracellular senile plaques composed of overproduced Aβ and intracellular neurofibrillary tangles consisting of hyperphosphorylated tau protein (Gouras, Olsson, & Hansson, 2015; Ramirez‐Rodriguez et al., 2011). The Aβ42 oligomer is recognized as the prime molecule in AD pathology and has been reported to induce synaptic disorder, including dendritic morphology abnormalities. In cultured neurons from the Tg 2,576 mice or neurons treated with Aβ from WT mice, the dendritic complexity is dramatically reduced compared to that in untreated WT cultures (Gouras et al., 2015). In rat organotypic slices, continuous overproduction of Aβ42 at dendrites or axons reduces the spine density and plasticity at nearby (~5 to 10 µm) dendrites (Wei et al., 2010). Dendrites are known to play an important role in synaptic plasticity; therefore, preventing the loss of dendritic complexity and dendritic spines caused by Aβ may be an effective strategy for reversing the synaptic disorder observed in AD. We recently reported that melatonin treatment in vivo partially rescued scopolamine‐induced dendritic spine loss, and melatonin had been suggested to protect against Aβ42‐induced inhibition of hippocampal LTP (Liu et al., 2013), indicating that melatonin may prevent Aβ42‐induced dendritic abnormalities. In this study, we found that melatonin was capable of restoring the loss of dendritic complexity and spines caused by Aβ42. Furthermore, we also found that a MT2‐selective agonist, IIK7, ameliorated the dendritic impairments, suggesting a specific role for MT2 activation in the protection of synaptic functions. Given that a loss of MT2 expression has been found in the AD brain and a reduction in MT2 expression was found in Aβ42‐treated neurons in this study, the preservation of MT2 activation might be a beneficial therapeutic strategy to treat the early symptoms of AD, such as synaptic loss.

Over the past decade, an increasing number of scientists have focused on the potential roles of epigenetic factors in the pathogenesis of AD because less than 3% of AD cases are familial (Ballard et al., 2011). As one of the important epigenetic regulators, miRNAs had been proven to be crucial for normal synaptic function (Hu & Li, 2017). Recently, the critical roles of miRNAs in AD have been well studied. The expression many miRNAs is altered in the AD brain at different stages, and many miRNAs are involved in the pathological changes of AD. Among them, miR‐125b is of particular interest for the following reasons. (a) miR‐125b is enriched in the brain and is critical to synaptic plasticity; (b) miR‐125b expression is upregulated in AD brains; (c) miR‐125b overexpression induces tau phosphorylation at multiple sites. In this study, we found deregulation of not only miR‐125b but also other miRNAs upon Aβ42 stimulation. However, MT2 activation only restored the level of miR‐125b but not the other miRNAs. By analyzing the 5′proximal promoter region of miR‐125b, we identified putative binding sites for transcriptional factors STAT3, C/EBPα, CDX2, and GATA1. It is known that the phosphorylation of STAT3 can be induced by cdk5 (Fu et al., 2004; Wen et al., 2008), an important kinase implicated in the AD pathogenesis, while the phosphorylation site is Ser727 but not Tyr705 here. In cultured neurons treated with Aβ, melatonin, or IIK7, only the alteration in C/EBPα activity was consistent with the change in miR‐125b expression. C/EBPα is enriched in neurons and is regulated by cAMP‐dependent signaling mechanisms (Kfoury & Kapatos, 2009), which are inhibited by MT2 activation. Through application of 8‐Br‐cAMP (a cAMP analog) and Rp‐cAMP (a cAMP inhibitor), we discovered that cAMP elevation led to activation of C/EBPα activity, which in turn resulted in miR‐125b over‐transcription. A previous study suggested that the activation of C/EBPα may regulate gene expression and consequentially has a role in the activation and/or proliferation of microglia following brain injury (Walton et al., 1998). Correspondingly, as the primary culprit in AD, Aβ, especially in its oligomer form, has been reported to impair neuronal functions, as well as dendritic complexity as shown here. Upon Aβ42 stimulation, the cAMP level is increased. As previously reported, MT2 activation induces cAMP inhibition. Here, we also found that both melatonin and IIK7 treatment suppressed the cAMP level and downregulated C/EBPα activity, as well as miR‐125b expression. Our results suggested a critical role of the cAMP/C/EBPα signaling pathway in the regulation of miR‐125b expression that is affected by Aβ and MT2 activation.

In addition, we also reported that activation of MT2 by IIK7 or melatonin preserved the protein level of GluN2A, a direct target of miR‐125b. In mice with genetic deletion of GluN2A, cell proliferation and differentiation in the dentate gyrus (DG) are intact. However, the total dendritic length and dendritic complexity in DG neurons located in the inner granular zone are dramatically decreased upon GluN2A deletion (Walton et al., 1998). Specifically, GluN2B‐containing *N*‐methyl‐D‐aspartate receptors (NMDARs) mediate Aβ42‐induced hTau‐dependent toxicity, whereas GluN2A‐containing NMDARs are involved in dendritic spine loss via the activation of caspase‐3 (Tackenberg et al., 2013). In our study, activation of MT2 by melatonin or IIK7 rescued the abnormalities in dendritic complexity and spines induced by Aβ42, as well as the upregulation of miR‐125b and loss of GluN2A. Application of miR‐125b fully blocked the protective effects of IIK7 to dendritic injuries induced by Aβ. Importantly, GluN2A mRNA is associated with fragile X mental retardation protein (FMRP) in the rodent brain and miR‐125b participates in FMRP regulation of GluN2A (Edbauer et al., 2010). Because FMRP expression is increased in the AD brain (Zhang et al., 2018), we propose the possibility of a synergistic effect of FMRP and Aβ in the downregulation of miR‐125b, which requires further study. Besides the GluN2A, many other identified downstream targets of miR‐125b might also be involved in the dendritic complexity regulation in AD. For example, miR‐125b blocks the expression of the phosphatases DUSP6 and PPP1CA as well as the anti‐apoptotic protein Bcl‐w, which in turn results in the tau hyperphosphorylation (Banzhaf‐Strathmann et al., 2014), the critical pathological changes that implicated in the dendritic abnormalities in AD (Hoover et al., 2010).

**Figure 6:**
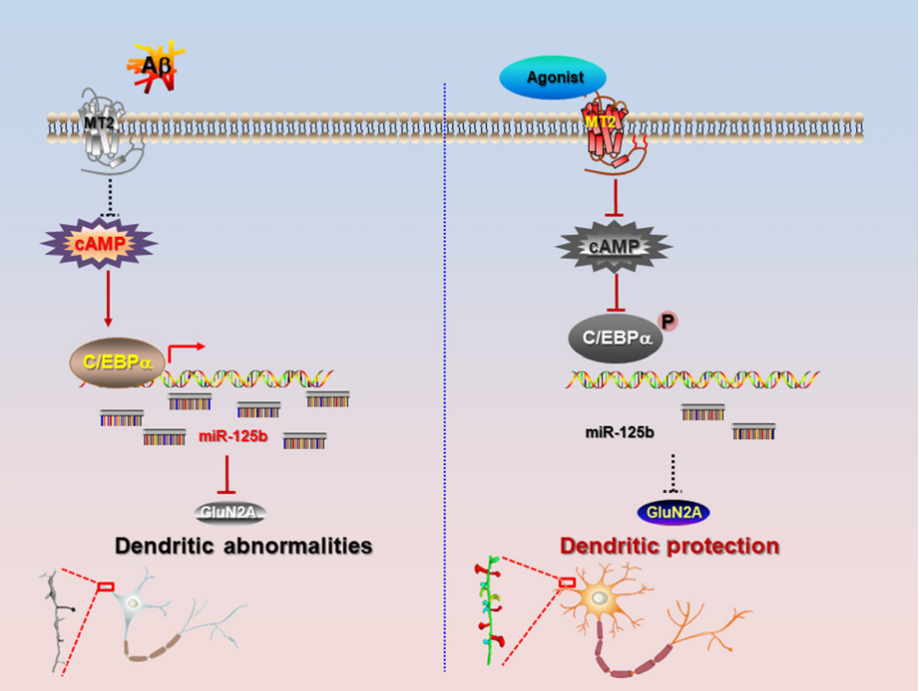
A working model for the neuroprotection of MT2 signal. In the AD brain, MT2 is downregulated and induces the upregulation of cyclic adenosine monophosphate (cAMP), which results in the activation of C/EBPα and upregulation of miR‐125b significantly. The increased miR‐125b post‐transcriptionally suppressed the expression of GluN2A and in turn leads to the dendritic abnormalities. Activation of MT2 or inhibition of miR‐125b is able to rescue those aberrant, as well as the learning/memory impairments

Overall, the study reported here demonstrated potential neuroprotective effects of MT2 activation on Alzheimer‐like dendritic impairments, including the loss of complexity and spines. We also revealed that miR‐125b, regulated by C/EBPα transcriptional factor, and its target, GluN2A, are involved in the underlying mechanism for the neuroprotective effect of MT2 activation (Figure 6), which could be a useful treatment for synaptic disorder in AD.

## 4 EXPERIMENTAL PROCEDURES

### 4.1 Reagents and antibodies

Please see Supporting Information Table S1 for details.

### 4.2 Generation of the C/EBPα‐shRNA‐expression cassette

C/EBPα (mouse, NM_007678)‐targeted sequence for knockdown was selected according to the Ambion web‐based criteria, followed by BLAST search analysis to avoid significant similarity with other genes. The sequences of the oligonucleotides are 5′‐ACCGGTGCGACGAGTTCCTGGCCGACTTCAAGAGAGTCGGCCAGGAACTCGTCGTTTTTTGGAAG‐3′; and 5′‐AATTCTTCCAAAAAACGACGAGTTCCTGGCCGACTCTCTTGAAGTCGGCCAGGAACTCGTCGCA‐3′. After annealed, the C/EBPα shRNA‐expression cassette was digested with the enzymes *Age* I and *Eco*R I and then cloned into the same sites in the lentiviral vector pLKD‐CMV‐eGFP‐U6‐shRNA.

### 4.3 Animals

Male APP/PS1 mice were purchased from the Jackson Laboratory (Bar Harbor, ME) (Stock#034829). Male APP/PS1 mice and their nontransgenic littermates were bred in the Experimental Animal Central of Tongji Medical College, Huazhong University of Science and Technology. All mice were housed under standard housing conditions with food and water ad libitum. Animal use and care was under the guidelines of the Animal Ethics Committee. Mice were maintained under a 12‐hr light on and 12‐hr light off schedule. Male mice were used throughout our experiment.

### 4.4 Analysis of dendritic complexity and spines

Sholl analysis was used to measure dendritic complexity as reported previously (Liu et al., 2017; Serita, Fukushima, & Kida, 2017). It was conducted and analyzed using the semi‐automatized Imaris software (Bitplane, Inc). The dendritic complexity index (DCI) was calculated for each neuron as we performed before. DCI = (Σ branch tip orders + # branch tips) × (total dendritic length/total number of primary dendrites). Dendritic spines were measured according to semi‐automatized protocol via Imaris software (Bitplane, Inc) based on spine‐head width and spine length.

### 4.5 Primary hippocampal neuron culture

The isolated embryonic hippocampal neurons (E18) were cultured as previously described (Liu et al., 2017). Briefly, the collected hippocampi were digested with 4 ml of 0.25% trypsin in D‐Hanks for 15 min and the digestion was stopped by adding at least 4 ml of the neuronal planting medium (10% fetal bovine serum in DMEM/F12). After centrifugation, resuspending and trituration with the planting medium, the cells were plated onto a 60‐mm plastic culture dish coated with poly D‐lysine. The neurons were incubated in the humidified incubator with 5% CO_2_ at 37°C for 2–4 hr, and the medium was replaced with Neurobasal medium supplemented with 2% B27 (maintenance medium). The maintenance medium was half volume replaced by fresh maintenance medium every 3 days.

### 4.6 Brain Stereotaxic injection

Mice were anesthetized using chloral hydrate (600 mg/kg, intraperitoneal). Holes were drilled above the CA1 field of the hippocampus (anterior/posterior = ±1.9 mm, medial/lateral = ±1.1 mm, dorsal/ventral = ±1.4 mm). Inhibitor of miR‐125b (2 μl) or lentivirus (2 μl) was bilaterally microinfused into the APP/PS1 or WT hippocampus via a cannula connected to a Hamilton (Reno, NV) microsyringe. The infusion rate was 0.2 μl/min, and the cannula was held in place for 10 min following completion of the infusion.

### 4.7 Morris water maze

Morris water maze was conducted to assess the spatial learning and memory as described previously (Hu et al., 2015; Wang et al., 2014). Briefly, the mice were trained for six consecutive days to find a platform hidden 1 cm under water using a stationary array of cues on the walls. A digital tracking device was connected to a computer and was used to track the movement of mice in the pool. On the 7th day, the escape latency of mice to reach the hidden platform was detected. On the 9th day, the hidden platform was removed. The latency to reach the place of platform and number of times mice cross the exact location of the platform were also recorded.

### 4.8 Electrophysiological recording

As previously described (Wang et al., 2018), for mEPSCs recording, the cultured neurons were treated with Aβ (1 μM), Aβ plus melatonin (500 μM), Aβ plus IIK7 (10 μM) at DIV 7 for 2 days. The whole‐cell patch clamp recordings were performed at DIV 9. Neurons were then placed in a recording chamber at room temperature and continually perfused at 2 ml/min with artificial cerebrospinal fluid containing (in mM): 125 NaCl, 5.4 KCl, 20 HEPES, 15 glucose, 1.2 MgCl_2_, 2 CaCl_2_ (pH 7.35, 300 mOsm). Patch electrodes (3–5 MG) were filled with an internal solution containing (in mM): 130 potassium gluconic acid, 12 KCl, 10 HEPES, 0.2 EGTA, 2 MgATP, 0.3 Li_2_GTP, 5 QX314 (pH 7.32, 287 mOsm). mEPSCs were measured with a gap‐free model and analyzed using Clamp fit 10.2 (Axon Instruments, Union City, CA, USA).

Brain slices (300 μm) were used for fEPSP recording as previously described (Wang et al., 2018). Slices were pre‐incubated in oxygenated artificial CSF at 32°C for 1.5 hr, transferred to a recording chamber perfused constantly with artificial CSF. A planar multi‐electrode recording setup (MED64, Alpha Med Sciences) was used to record the fEPSP. The fEPSPs were recorded from the CA1 neurons of the Schaffer collateral pathway as the stimulating electrode in CA3. Input–output relationship was generated by delivering 10–100‐µA electrical stimulation, and the amplitude of the peak fEPSPs was measured. The stimulation intensity evoking the fEPSP with a magnitude of 40% of the maximum response was chosen. LTP induction protocol consists of three trains (inter‐train intervals of 30 s) of 100‐Hz stimulus which lasted for 1 s was applied after a stable baseline of 30 min, and the field potential response for 2 hr after the tetanus was recorded. Electrophysiology experiments were performed blinded.

### 4.9 Generation of Aβ42 oligomer

Synthetic Aβ‐(1–42) peptide (Chinapeptides, Shanghai, China) was prepared as instructions (Wang et al., 2018). Briefly, the peptide was dissolved in 100% hexafluoroisopropanol (HFIP) to 1 mM, and then, HFIP was removed by vacuum, resuspended with Me_2_SO (DMSO), and then further diluted by F12 (without phenol red) culture medium to 100 μM and incubated for 24 hr at 4°C. Then, the solution was aliquoted and stored at −80°C until use. Before usage, the solution was centrifuged at 16,000 *g* for 20 min to remove the fibril forms of Aβ1–42. Then, the dissolved oligomeric Aβ1–42 that was present in the supernatant was used for further experiments. Concentration of Aβ42 oligomer is calculated by using oligomeric Amyloid‐beta ELISA Kit assay. The work concentration of Aβ42 oligomer is 1 μM.

### 4.10 cAMP ELISA

The cAMP levels in the cell lysis were assayed according to the manufacturer's procedure (Cat No: KB1005A, Boyao, Shanghai, China). The cell lysis will be added to the microtiter plate wells that coated by purified human cAMP antibody, and then, the HRP‐labeled cAMP antibody will be added to the wells to form an antibody/antigen/enzyme‐antibody complex. After washing by washing buffer for 1 min × 5 times, then the 3, 3′, 5, 5′‐tetramethylbenzidine substrate solution was added. The HRP enzyme‐catalyzed reaction will be stopped by the addition of a sulfuric acid solution at 15 min later, and the color change is measured spectrophotometrically at 450 nm. The concentration of cAMP in the samples is then determined by comparing the O.D. value of the samples to the standard curve.

### 4.11 Immunofluorescence

The primary hippocampal neurons on days in vitro (DIV) 9 or DIV 21 were fixed in situ for 15 min by 4% PFA (pH 7.2, stored at 4°C). The cell membrane was penetrated with 0.5% Triton in PBS for 15 min and washing with PBS for 3 times. After blocking with 3% BSA at room temperature for over 30 min, the primary antibodies (as list in Supporting Information Table S1) were added and incubated at 4°C overnight. Then, the red or green fluorescent secondary antibodies were added to the slices and followed by PBS washing for three times. Hoechst 33,258 was added to visualize the nuclear. Images were captured by using a Zeiss LSM780 laser confocal microscope (Zeiss, Jena, Germany).

### 4.12 RNA extraction and Q‐PCR for miRNAs

Total RNA from DIV 9 hippocampus neurons treated with indicated reagents was extracted by TRIzol reagent, and 1 μg RNA was reverse transcribed. A miRNA isolation kit (Tiangen, Beijing, China) was used for microRNA extraction. qRT–PCR was performed on an ABI StepOne Plus system (Applied Biosystems) by using SYBR Green Premix Ex Taq (Takara). Total reaction volume is of 10 μl containing 0.5 μl cDNA (100 ng/μl), 1 μl of each 2 μM primer (300 mM each), 5 μl SYBR Green, and 2.5 μl RNase/DNase‐free sterile water. Each master mix was run in triplicate to be parallel control. Cycle conditions were set as follows: initial template denaturation at 95°C for 1 min, followed by 40 cycles of denaturation at 95°C for 5 s, combined primer annealing at 60°C for 30 s, and elongation at 72°C for 30 s. This cycle was followed by a melting curve analysis, ranging from 60 to 95°C, with temperature increases by steps of 0.5°C every 10 s. Primers for miRNA detection are listed in Supporting Information Table S2.

### 4.13 Western blotting

The Western blotting was carried out by a well‐established procedure in our laboratory (Xiong et al., 2015). Briefly, the cell extracts prepared from cultured neurons or hippocampal tissues were separated by SDS‐PAGE gel (10%), and then, the proteins were transferred onto nitrocellulose membrane (Amersham, Piscataway, NJ, USA) for 1 hr by using the transfer apparatus (Bio‐Rad, Berkeley, CA, USA). After blocking with 3% milk for at least 30 min at 25°C, the membranes were incubated at 4°C with primary antibodies overnight. The blots were probed by using IRDye 800CW‐conjugated secondary antibody and visualized by infrared fluorescence imaging. The intensity of the protein bands was quantified by using the Odyssey software (Li‐Cor Bioscience, Lincoln, NE, USA). BCA kit (Pierce, Rockford, IL, USA) was used to quantify the protein concentration.

### 4.14 Statistical analysis

Data were analyzed using the SPSS 11.0 statistical software (SPSS, Inc., Chicago, IL, USA), and the one‐way ANOVA procedure followed by Tukey's multiple comparisons tests was used to determine the differences between the groups if not specifically described. Data are presented as means ± *SEM*.

## CONFLICT OF INTEREST

The authors declare no competing financial interests.

## AUTHOR CONTRIBUTIONS

L.‐Q.Z. and D.L. conceived and designed the studies and wrote the paper. H.T. performed confocal imaging and 3D reconstruction. M.M. and Y.W. carried out injections and behavior tests. M.‐F.D., F.H., and H.‐Z.H. performed both in vivo and in vitro electrophysiological recordings. H. A. carried out cell counting and Western blot. D.‐Q.W. contributed to the diagram of working model. L.‐T.Z. performed the primary neurons culture. All authors contributed to the data analysis and presentation in the paper.
