# Supplementary material for: Activation of MT2 receptor ameliorates dendritic abnormalities in Alzheimer’s disease via C/EBPα/miR‐125b pathway
Source: Aging Cell. 2019 Feb 1;18(2):e12902. doi: 10.1111/acel.12902 (PMC6413662; doi:10.1111/acel.12902)
Supplement: Supplementary file 1 [file ACEL-18-e12902-s001.docx]

**Activation of MT2 receptor ameliorates dendritic abnormalities in Alzheimer’s disease via C/EBPα/miR-125b pathway**

***Supplementary information***

***
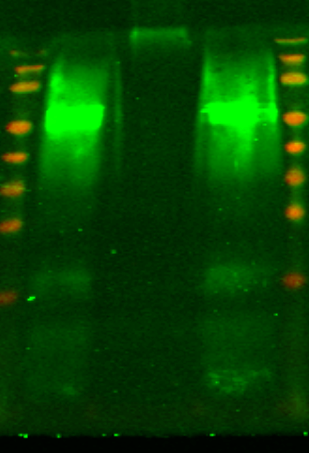
***

**M**

**1**

**2**

**M**

**3**

**17**

**34**

**fibrils**

**170**

**KDa**

**large oligomers**

**monomers**

**Supplementary Figure 1.** Assessments of Aβ42 under oligomer and fibril forming conditions. Western blot analysis of Aβ42 oligomers and fibrils separated on a 10% SDS/PAGE gel and probed with the monoclonal antibody 4G8. Lane 1 shows Aβ42 oligomers formed at 4℃ after 24 h, Lane 2 shows Aβ42 fibrils formed at 37℃ after 1 week and Lane 3 shows Aβ42 oligomers subjected to freeze and thaw once. Molecular masses are indicated as KDa. M means marker.

******

(b)

(a)

**Supplementary Figure 2.** Cell viability analysis after Aβ42 oligomer treatment. (a) Mouse primary hippocampal neurons at DIV 7 were treated with 1 μM Aβ42 oligomer or the Scrambled for 48h, followed by the viability analysis by using CCK-8 kit. N=4-5. (b) Cell survival analysis was assessed by CCK-8 assay when treated with variant Aβ42 oligomer (from 1 μM to 6 μM) or scrambled petpide. ^*^*p*<0.05, ^**^*p* < 0.01 (versus Scramble); one-way ANOVA, Tukey’s multiple comparisons test; ^#^*p* < 0.05, ^##^*p* < 0.01 (versus 1 μM); one-way ANOVA, Tukey’s multiple comparisons test, N=4-5. Data are presented as the mean ± SEM.

**
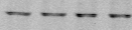

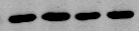
**

**MT1**

**DM1A**

**KDa**

**40**

**55**

**Scramble**

**Aβ**

**Aβ+melatonin**

**Aβ+IIK7**

(a)

(b)

**Supplementary Figure** 3**. MT1 expression is not altered upon the treatment of Aβ42 or Aβ42+melatonin or Aβ42+IIK7.** Cell lysates from mouse primary hippocampal neurons were treated with Aβ, Aβ+melatonin, Aβ+IIK7, or the scrambled peptide at DIV 7. 48 hours later, MT1 protein level is evaluated by western blot (a) and subjected to quantification analysis (b). Data are presented as the mean ± SEM. Results repeated at least three independent experiments. N=4-5 per group.

**c-fos**

**GAPDH**


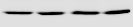


**Scramble**

**Aβ**

**Aβ+melatonin**

**Aβ+IIK7**

**KDa**

**70**

**34**


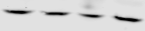


(b)

(a)

**Supplementary Figure** 4**. Activation of the MT2 receptor rescues c-fos level.** The mouse primary hippocampal neurons were treated with Aβ, Aβ+melatonin, Aβ+IIK7, or the scrambled peptide at DIV 7. 48 hours later, the cell lyses were prepared for the western blot by using c-fos antibody (a) and subjected to quantification analysis (b). ^**^*p* < 0.01 (versus Scramble); one-way ANOVA, Tukey’s multiple comparisons test; ^#^*p* < 0.05, ^##^*p* < 0.01 (versus Aβ); one-way ANOVA, Tukey’s multiple comparisons test, N=4-5 per group. Results repeated at least three independent experiments. Data are presented as the mean ± SEM.

**
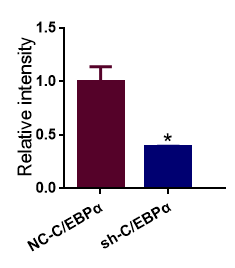
**

(b)


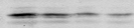


**C/EBPα**

**KDa**

**43**

**sh-C/EBPα**

**NC-C/EBPα**

**55**


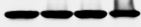


**DM1A**

(c)

(a)

**Supplementary Figure** 5**. Silencing of C/EBPα blocks the Aβ42 induced miR-125b up-regulation.** (a-b) Neuro2a neuroblastoma cells were transfected with control shRNA (NC-C/EBPα) or C/EBPα-shRNA (sh-C/EBPα). 72 hours later, the cell lyses were collected and the C/EBPα expression level were evaluated by western blot (a) and subjected to quantification analysis (b). Histograms show mean ± SEM after normalized to DM1A levels. ^*^*p* < 0.05 (versus control; Student’s t test; N=3-5 per group). (c) Neuro2a neuroblastoma cells were transfected with control shRNA (NC-C/EBPα), C/EBPα-shRNA (sh-C/EBPα) or lipofectamine® 3000 reagent alone, 24 hours later, Aβ42 oligomer and its scrambled peptide were introduced into the above wells for 48 h, nominated as Scramble, Aβ, Aβ+NC-C/EBPα, Aβ+sh-C/EBPα. Total RNA was extracted, followed by reverse transcription as described previously. Relative miR-125b expression level from the above cDNA was quantified by real time PCR. ^*^*p* < 0.05 (versus scramble), ^&^*p* < 0.05 (versus Aβ+NC-C/EBPα); one-way ANOVA, Tukey’s multiple comparisons test; N=4-5 per group. Data are presented as the mean ± SEM.

**
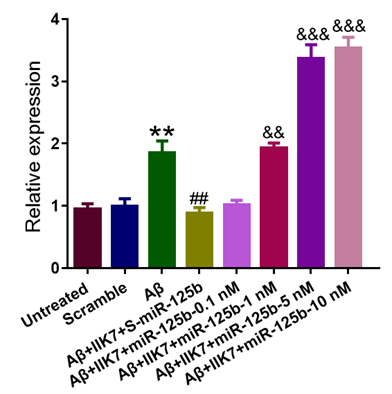
**

(a)

**Supplementary Figure** 6**. The relative expression levels of miR-125 upon the different treatments.** Mouse primary hippocampal neurons at DIV 7 were treated with scrambled peptide (Scramble), Aβ, Aβ plus IIK7 plus the scrambled control for miR-125b (Aβ+IIK7+S-miR-125b), Aβ plus IIK7 plus miR-125b mimics (from 0.1 nM to 10 nM) as indicated for 2 days. Total RNA was extracted, followed by reverse transcription as described previously. Relative miR-125b expression level from the above cDNA was quantified by real time PCR. ^*^*^*^p*<0.01 (versus Scramble); ^##^*p*<0.01 (versus Aβ); ^&&^*p*<0.01, ^&&&^*p*<0.001 (versus Aβ+IIK7+S-miR-125b); one-way ANOVA, Tukey’s multiple comparisons test, N=4-5. Results repeated at least three independent experiments. Data are presented as the mean ± SEM.

**
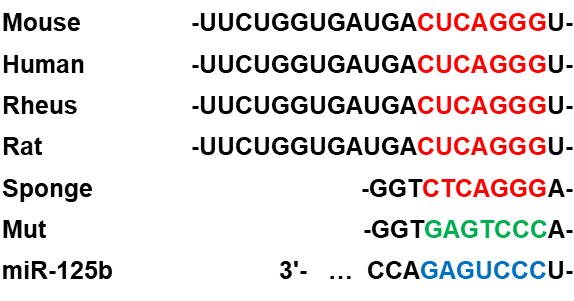
**

**GluN2A**

(a)

(b)

**
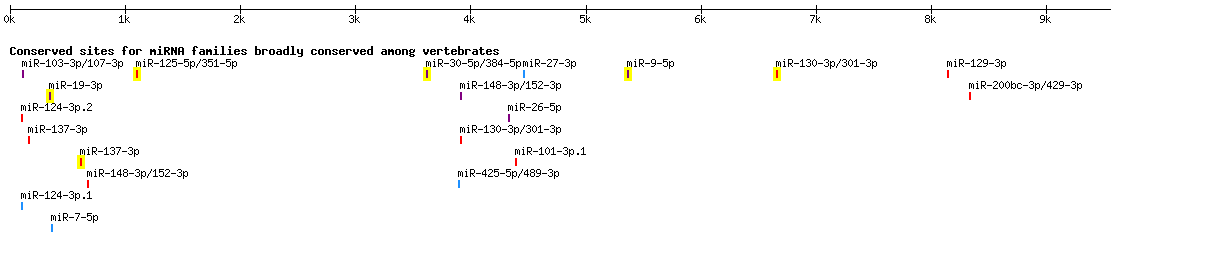
**

(c)

**
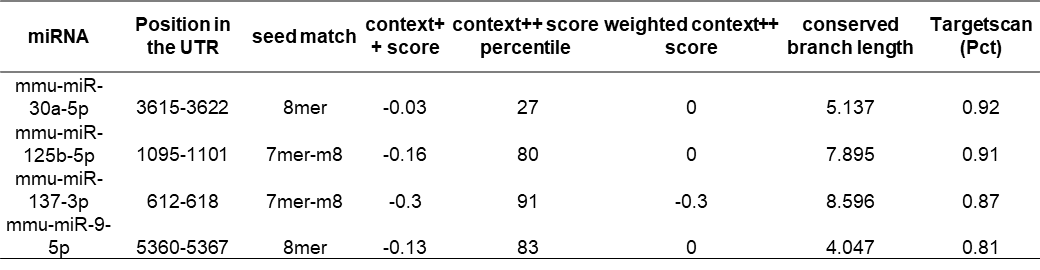
**

**Supplementary Figure** 7**. Analysis of 3’UTR of GluN2A with miR-125b.** (a) Alignment of the 3′UTR sequence of GluN2A in four mammalian species with miR-125b as well as its sponge sequence (repeated 6 times) and the mutant (repeated 6 times). (b) Predicted microRNAs binding in GluN2A 3′UTR by TargetScan (v7.2). (c) Separating conserved and highly conserved miRNAs that targeting the 3’UTR of GluN2A based on the *P*_CT_ values (>0.8) predicted by TargetScan (v7.2).

**
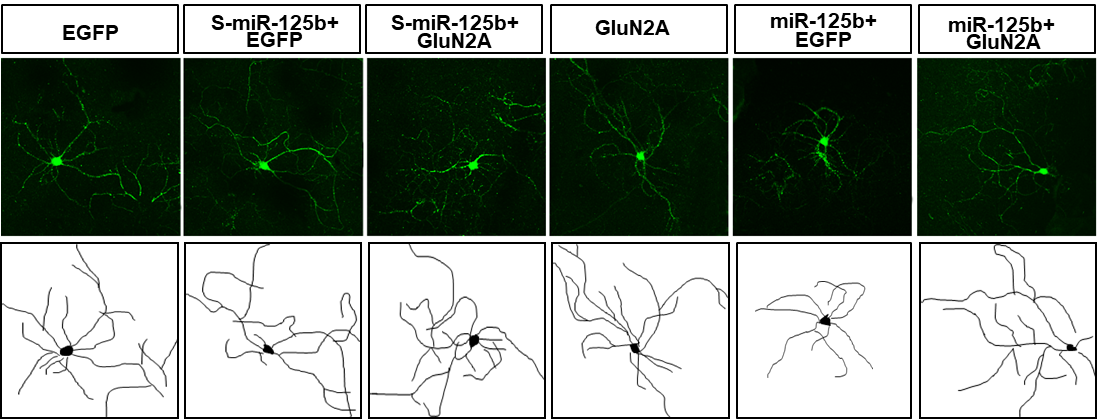
**

**Supplementary Figure** 8**. miR-125b disrupts dendrite integrity in a GluN2A-dependent fashion.** Mouse primary hippocampal neurons at DIV 7 were treated with EGFP, S-miR-125b+EGFP, S-miR-125b+GluN2A, GluN2A, miR-125b+EGFP or miR-125b+GluN2A for 48 hours. Representative confocal images (upper) and their reconstructions (lower) are shown. Scale bar=40 μm. Results repeated at least three independent experiments.

(b)

(a)

**Supplementary Figure** 9. **Relative expression of miR-125b upon different manipulations.** (a) Mouse primary hippocampal neurons at DIV 7 were treated with scrambled control for miR-125b (S-miR-125b) or miR-125b mimics (miR-125b-mimic) for 2 days. Total RNA was extracted, followed by reverse transcription as described previously. Relative expression of miR-125b from the above cDNA was quantified by real time PCR. ^**^*p*<0.01 (versus S-miR-125b); Student’s t test; N=4-5. (b) Total RNA from Mouse primary hippocampal neurons treated with scrambled control (S-I-miR-125b), inhibitor of miR-125b (I-miR-125b), or the mutant (Mut-I-miR-125b) at DIV 7 were collected 2 days later (DIV 9), followed by reverse transcription as described previously. Relative miR-125b expression from the above cDNA was quantified by real time PCR. *^**^p*<0.01 (versus S-I-miR-125b); ^##^*p*<0.01 (versus Mut-I-miR-125b); one-way ANOVA, Tukey’s multiple comparisons test, N=4-5. Data are presented as the mean ± SEM.

**Supplementary Table 1. Antibodies and reagents**

| **Antibodies & Reagents** | **Catalog no.** | **Source** | **MAb/RAb** | **Dilution** |
| --- | --- | --- | --- | --- |
| anti MAP2 | M4403 | Sigma | M | 1:300 for IHC/IF |
| Anti melatonin Receptor 1B | bs-0963R | Beijing Biosynthesis Biotechnology | R | 1:300 for IHC/IF, 1:500 for wb |
| anti-GluN2A | 19953-1-AP | Proteintech | R | 1:1000 for wb |
| anti-STAT3 | 8768S | Cell Signaling Technology | R | 1:1000 for wb |
| anti-STAT3(phospho Y705) | ab30646 | abcam | R | 1:500 for wb |
| anti-CEBPα | 18311-1-AP | Proteintech | R | 1:500 for wb |
| anti-Phospho-CEBPα (Ser21) | bs-3060R | Beijing Biosynthesis Biotechnology | R | 1:500 for wb |
| anti-GATA1 | 10917-2-AP | Proteintech | R | 1:500 for wb |
| anti melatonin Receptor 1A | bs-0027R | Beijing Biosynthesis Biotechnology | R | 1:500 for wb |
| anti c-fos | 26192-1-AP | Proteintech | R | 1:1000 for wb |
| anti-alpha Tubulin antibody（DM1A） | 66031-1-Ig | Proteintech | M | 1:1000 for wb |
| GAPDH | 60004-1-Ig | Proteintech | M | 1:5000 for wb |
| Alexa Fluor 488 goat antimouse IgG (H+L) | A-11001 | Invitrogen | M | 1:1000 for IF |
| Alexa Fluor 555 goat anti-rabbit IgG (H+L) | A32732 | Invitrogen | R | 1:1000 for IF |
| Hoechest | H3570 | Invitrogen | / | 1:5000 for IF |
| odessey goat anti rabbit | 926-3211 | biosciences | R | 1:10000 for wb |
| odessey goat anti mouse | 96-32210 | biosciences | M | 1:10000 for wb |
| β-Amyloid (1-42), Human | 40700011526 | chinapeptides | / | 1μM |
| Melatonin | M5250 | Sigma | / | 500μM |
| IIK7 | I5531 | Sigma | / | 100μM |
| 4P-PODT | SML1189 | Sigma | / | 100μM |
| 8-Br-cAMP | B7880 | Sigma | / | 2mM |

**Supplementary Table 2. Primers for miRNA detection**

| **Name** | **Primer sequence** |
| --- | --- |
| miR-125b | TCCCTGAGACCCTAACTTGTGA |
| miR-134 | TGTGACTGGTTGACGAGAGGG |
| miR-135a | TATGGCTTTTTATTCCTATGTGA |
| miR-138 | AGCTGGTGTTGTGAATCAGGCCG |
| miR-29a | TAGCACCATCTGAAATCGGTTA |
| miR-29b | TAGCACCATTTGAAATCAGTGTT |
| miR-132 | TAACAGTCTACAGCCATGGTCG |
| miR-124 | TAAGGCACGCGGTGAATGCC |
| miR-34 | TGGCAGTGTCTTAGCTGGTTGT |

**Supplementary Table 3. List of transcriptional factors that regulates miR-125b**

| **TFs** | **influence on miR125b** | **Reference** |
| --- | --- | --- |
| STAT3 | upregulation | Dai CY et al., 2016 |
| C/EBPα | upregulation | Vargas Romero P et al., 2015 |
| CDX2 | upregulation | Lin KY et al., 2011 |
| GATA1 | unknown | Lin KY et al., 2011 |
